# The effect of Pap smear screening on cervical cancer stage among southern Thai women

**DOI:** 10.1038/s41598-019-52607-6

**Published:** 2019-11-15

**Authors:** Li Niu, Shama Virani, Surichai Bilheem, Hutcha Sriplung

**Affiliations:** 1 School of Basic Medical Sciences, Jiujiang University, Jiujiang, Jiangxi China; 2 Epidemiology Unit, Faculty of Medicine, Prince of Songkla University, Hat Yai, Songkhla Thailand

**Keywords:** Cancer screening, Health policy

## Abstract

Our study aimed to investigate the effect of Pap smear screening on stage at diagnosis of cervical cancer in a heterogeneous population of Thai women. Data was merged from the population-based cancer registry and screening registry based on unique identification numbers from 2006 to 2014. Patients being screened had lower odds to be diagnosed at late stage. After adjustment, married women had reduced risk of late stage cancer compared to single women. Muslim women had almost twice the risk of being diagnosed late stage compared to Buddhist women. The odds of being diagnosed at late stage decreased with increased number of screening. The probability of being diagnosed at late stage increased rapidly among females aged 40 to 55 years. Pap smear screening is a protective factor in diagnosis of late stage cervical cancer. Patients were more likely to be diagnosed at early stage with more frequent screening. For future screening programs, it will be beneficial to shorten screening intervals and take more concern for vulnerable population: women aged between 40 and 55 years, and women who are single or Muslim.

## Introduction

Cervical cancer ranks the fourth most common cancer worldwide (570,000 cases and 311,000 deaths in 2018), accounting for 7.5% of all female cancer deaths. Nearly 90% cervical cancer deaths occur in the less developed regions^1^. In Thailand, cervical cancer has been a leading cancer for decades with an estimated 24.7 per 100,000 age-standardized incidence rate in the period of 1998–2000^2^, then dropped down to 14.0 per 100,000 in the period 2010 to 2012^3^.

The Thai Ministry of Public Health initiated a screening program in 2002 for women aged from 35 to 60 years at 5-yearly intervals^4^ and later extended it to women aged from 30 to 60 years^5^. The coverage of the screening, as evaluated by the Health Intervention and Technology Assessment Program, was around 68% by 2009–2010^6^. Two national surveys done in 2007 and 2009 reported a coverage of 46.3% and 59.7%, respectively^7^. Variation in coverage across regions existed with Bangkok Metropolitan being the poorest at around 44%. Pap smear and visual inspection with acetic acid are screening methods covered under the universal health care (UHC)plan, while the human hapillomavirus virus vaccine is not covered by UHC due to its unaffordable price^7^.

Cervical cancer incidence dropped after countrywide screening^8^. The incidence of cervical cancer declined by 4.7% per year from just give the number at the peak in 1998–2000 in Songkhla^9^. However, screening rates for cervical cancer were not optimal among several groups of women: less or non-educated, poor, young, unmarried, and non-Buddhist women^7^. Furthermore, the extent to which Pap smear screening could affect the diagnosis of late stage cervical cancer lacked empirical evidence.

This study examined the effect of repeated Pap screening on cervical cancer diagnosis, as well as other risk factors, including residence, religion, marital status and age in at-risk neighborhoods.

## Results

Propensity scores were calculated based on selected covariates (marital status, age, religion and hospital level), to make the screened and not screened groups more comparable. The proportion of each covariate in the 2 groups became similar after propensity scores matching. Among not screened women, 58(12.7%) were Muslim whereas 16(17.6%) were Muslim among screened women (Table 1).

**Table 1:** Comparison of matched samples.

	Not screened	Screened	p
Marital = Married and Divorced (%)	415 (91.2)	83 (91.2)	1.00
Age (mean(sd))	48.29 (7.08)	48.52 (6.70)	0.78
Religion = Islam (%)	58 (12.7)	16 (17.6)	0.29
Hospital level			0.60
Secondary	8 (1.8)	1 (1.1)	
Tertiary	54 (11.9)	14 (15.4)	
Super tertiary	393 (86.4)	76 (83.5)	

Among screened women, 59(64.8%) were diagnosed with early stage cancer, while only 202(44.4%) were diagnosed at early stage among screened women (Table 2). The odds of a screened woman getting late-stage diagnosis were 0.43 times lower compared to a not screened woman.

**Table 2:** Distribution of Pap smear screening and cancer stage at Diagnosis.

Pap smear screening	Cancer stage			Chi-squared test	p-value
	Early stage	Late stage	Total	11.89	<0.001
Not screened	202 (44.4%)	253 (55.6%)	455		
Screened	59 (64.8%)	32 (35.2%)	91		

A generalized linear model (GLM), which assumed a linear association between screening and diagnosis, was applied to get the probability of being diagnosed at early or late stage based on multiple independent variables. After the model selection (see methods), the best model included five independent variables (screening frequency, marital status, age, religion and hospital level). The results showed that not being screened, being single, older age, and being Muslim had higher odds of being diagnosed at late stage (Table 3).

**Table 3:** Probability of being diagnosed at late stage.

	Crude OR (95%CI)	Adj. OR (95%CI)	P(Wald’s test)	P(LR-test)
No. of Screening (cont. var.)	0.65 (0.5,0.83)	0.61 (0.47,0.79)	<0.001	<0.001
Married and Divorced vs Single	0.77 (0.42,1.40)	0.50 (0.26,0.99)	0.045	0.042
Age: ref. = (34,40]				<0.001
(40,45]	0.93 (0.53,1.65)	1.20 (0.65,2.21)	0.555	
(45,50]	1.63 (0.92,2.90)	2.1 (1.13,3.87)	0.018	
(50,55]	2.96 (1.62,5.39)	3.94 (2.06,7.53)	<0.001	
(55,60]	2.7 (1.49,4.88)	3.55 (1.88,6.72)	<0.001	
Islam vs Other	1.49 (0.90,2.45)	1.85 (1.08,3.17)	0.025	0.023
Tertiary hospital and above vs below tertiary level	9.05 (1.12,72.84)	11.18 (1.34,93.43)	0.026	0.005

Log-likelihood = −347.2802.

No. of observations = 546.

The Adj.OR is adjusted for age, marital status, religion, hospital level.

**Figure 1:**
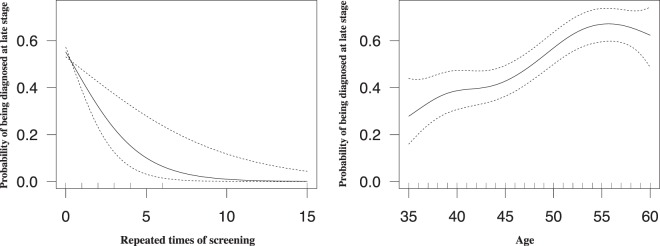
Smoothed graph of repeated times of screening and age. The curve is smoothed by the technique of thin plate splines, representing the change of the probability of being diagnosed at late stage.

Generalized additive model regression (GAM) was used to highlight nonlinear trends in the data. All applicable continuous variables were smoothed using thin plate splines to separate trends from noise. The y-axis was transformed to be interpreted as the logarithm of the ‘odds’ to the probability of being diagnosed at late stage. The GAM revealed hidden non-linear trends of disease stage with age at diagnosis and repeated time of screening: the probability of being diagnosed at late stage increases rapidly among female aged from 40 to 55, as well as being screened from 0 to 5 times, the probability of being diagnosed at late stage dropped over 15% from 3-time to 5-time screening (Fig. 1).

The result of the GAM was similar to the GLM (Table 4). The deviance explained by generalized additive model was 8.74%. Analysis of variance showed that the residual deviance and the Akaike information criterion of the generalized additive model was less than generalized linear model, illustrating the better fit of the generalized additive model fit (Table 4).

**Table 4:** Results and comparison of generalized linear model and generalized additive model.

	Dependent variable: cancer stage	
	Coefficient of GLM	Coefficient of GAM
Repeated time of screening	−0.478*** (0.133)	Smoothed term
Marital status (married and divorced)	−0.690** (0.334)	−0.686** (0.338)
Age	0.066*** (0.013)	Smoothed term
Religion (Islam)	0.597** (0.271)	0.590** (0.272)
Hospital level (Tertiary and above)	2.422** (1.075)	2.398** (1.080)
df	6.000	8.488
AIC	715.834	715.487
Residual deviance	703.83	698.51
R-sq (adj)		0.089
Deviance explained		7.59%

Note: *p < 0.1; **p < 0.05; ***p < 0.01.

## Discussion

Cancer stage at diagnosis is a critical determinant of cancer outcomes and is directly associated with survival in cancer patients^10^. Previous studies showed that women rarely or never screened were more likely to be diagnosed at late stage than women undergoing routine screenings^11–15^. Our study showed that patients were more likely to be diagnosed at an early stage with more frequent screening.

Studies in the past indicated that various disparities prevented women from being diagnosed at an early stage. A study from Florida indicated that elderly, unmarried, and uninsured women are more likely to be diagnosed late stage^16^. One study conducted in three American cities showed that residence in less developed neighborhoods tended to cause late-stage cancer diagnosis^17^, as well as physician characteristics, such as being screened before, or having visited doctors in the past 3 years which was revealed in a retrospective cohort study in Canada^18^.

Our analysis also validated that elderly and unmarried women had higher odds of getting a late stage diagnosis in Thailand. Moreover, women aged between 40 and 55 years were the most vulnerable population to late stage diagnosis. Possible explanations are that women ages 40 and 55 years are at a high risk for late stage cervical cancer because of having a sexual partner; unmarried females might get less social support or fear the loss of virginity or think of cervical cancer as a sexually transmitted disease which might prevent them from entering the clinic for screening.

Religious and cultural beliefs, especially those valuing modesty and premarital virginity, contribute to reluctance to seek health care^19^. In our study, Muslim women had a higher chance of getting a late stage diagnosis. Many Muslim women face challenges in obtaining adequate health care due to family pressures, especially from their husband^20^; they may resist screening practices that threaten their cultural and religious values. Additionally, Asian people consider cancer screening as a response to symptoms instead of tests to prevent the development of symptoms^21^.

The latest recommendation is to screen women with Pap tests every 3 years according to our findings and the recommendation made by the United States Preventive Services Task Force and American Cancer Societies^22^. Our findings show that more frequent screening might decrease the number of women diagnosed at late stage. More frequent Pap smear screening should be provided for high-risk group, such as unmarried, women aged 40 to 55 and Muslim women. Reminder letters, texts through mobile phones or door-to-door visit by community health workers might be good ways to notify the high-risk group to attend the screening on time.

Despite the better outcome of 3-year interval screening, many countries including Thailand used the 5-year interval due to the limited financial budget and human resources. Moreover, in 2017, Thailand government has initiated free HPV vaccination for Grade 5 students^23^, which is another way to prevent cervical cancer under the 5-year interval screening. Thus, we suggest a Pap smear screening program with 3-year interval when there are enough health workers and money for the whole society.

Our study had some limitations. First, registry data and screening data were not well matched leading to a limited number of cases available for this analysis; Second, several important independent variables were not included: socio-economic status, occupation, education, and age at birth of first child. Therefore, our findings may not be generalized to all women in Thailand. However, this is the first study to combine a screening database with a cancer registry in Thailand to identify factors that contribute to reduced screening and how it can affect cancer diagnosis. Also, propensity score matching was proved to be an effective way to link two separate databases. Lastly, generalized additive model revealed nonlinear trends that were important for particular age groups and these could not be detected by the commonly used generalized linear model.

## Methods

### Region

Songkhla province is located in southern Thailand with a population of 1,424,230 (25% Muslims)^24^. Although the estimated age-standardized incidence rate dropped from its peak 20.6 in 1999 to 14.0 per 100,000 in the period 2010 to 2012^3^, cervical cancer still ranks the second among common female cancer of Songkhla. The organized Pap smear screening program has started since 2004 in Songkhla^9^.

### Cancer registry and screening

The Songkhla registry covers sixteen districts in southern Thailand. Cancer cases have been collected from 23 sources including community and private hospitals, also the population registration office. Undetected cases still existed in remote villages due to poor access to health facilities and the utilization of traditional Thai medicine in lieu of health care services^25^.

From 1989–2014 Songkhla Cancer Registry data, 3,317 cervical cancer cases were selected by ICD-10 codes (C53.X for invasive cancer and D06.9 for carcinoma *in situ* of the cervix uteri). Cervical cancer screening data from 2001–2016 was provided by Songkhla Provincial Health Office. There were 114,222 persons screened with 208,039 times (visits).

### Data management

Although screening started in 2002, the cervical cancer screening database used before 2006 had a different data structure. Therefore, data from both the cancer and screening registries were only able to be merged based on unique registry numbers from 2006 to 2014 as this was the time period with the most complete variables needed for the analysis. Prior to 2006, missing data prevented any informative analysis.

Due to the nature of the cancer registry, only invasive cancers were included in this analysis. The cancer registry intends to collect invasive cancers only. If precancerous lesions were identified in the cancer registry, the data was considered incomplete as the invasive cancer was likely not collected. Including this information would bias our analyses as it would seem that the *in situ* cases did not progress to invasive carcinomas, although they likely did and were just not captured by the registry.

**Figure 2:**
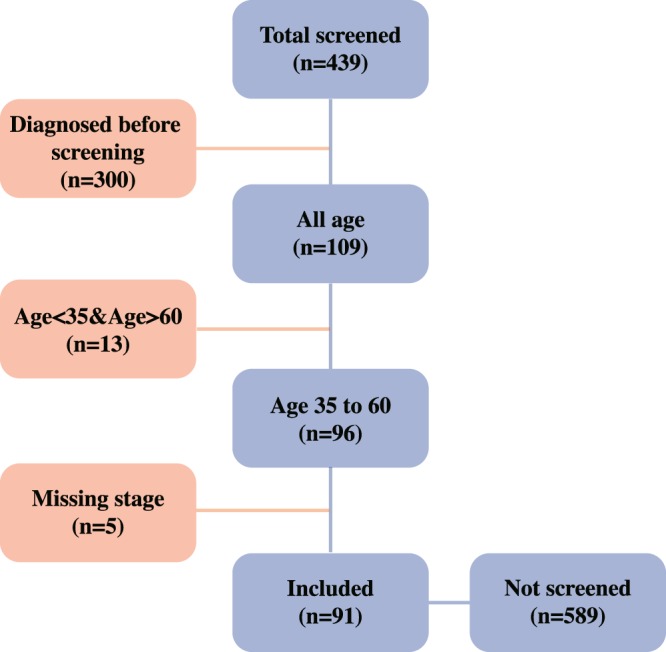
Study sample inclusion process.

Only 5 patients had unknown stage, and were excluded to only include those with complete stage information. We included 680 women for this retrospective population-based study. Of these, 91 women aged from 35–60 had their screening before diagnosis, and 589 had a cancer diagnosis but no screening history (Fig. 2).

A propensity score is the probability of a unit (e.g., person, hospital, department) being allocated to a specific treatment given a group of observed covariates. Selection bias can be reduced by equating groups based on these covariates. The propensity score was estimated by running a logit model where the outcome variable is a binary variable indicating the stage of cervical cancer. For the matching, covariates that were related to both the screening and outcome variables were included. The R package “MatchIt” was used for estimating the propensity score and then matches observations based on the method of choice (“nearest” in this case). After matching, 91 for screening group and 455 for no screening group were included.

### Data analysis

The stages of cervical cancer were tagged from 1 to 4. Stage 1—The cancer is contained within the cervix; Stage 2—The cancer reaches out of the cervix to the surrounding tissues; Stage 3—The cancer spreads outside the surrounding area of the cervix; Stage 4—This stage is advanced cervical cancer. Stage 1 was assigned to early stage, the rest as late stage.

Pap smear screening was categorized in two ways: binary variable (1 for being screened; 0 for not being screened) and count variable (0, 1, 2 to N times of being screened). Other independent variables included religion, age, marital status, hospital level, list all variables. Women aged 30–60 years were the target of the national screening program and therefore, the focus of this analysis. The first 5-year age group was omitted from the calculation as unstable estimates of the risk might occur in counting cases first entering the screening process by calendar year.

Data management and description analysis was done by using R packages including “epicalc”, “plyr” and “reshape2”. Logistic regression was conducted by using R package “ice”. Inference was made based on the chi-square test in univariate analysis. P-value less than 0.05 suggests there is a significant difference among different categories. The 95% confidence interval and p-value were calculated in the logistic regression model. The likelihood ratio test and Wald’s test were used to test the statistical significance of the variable in the logistic regression model. A p-value less than 0.05 suggests the statistical significance of a variable in the model. All statistical analysis was conducted using R software version 3.5.2.

A generalized additive model was conducted by using R package “mgcv”. Generalized additive models are generalized linear models in which the linear predictor depends linearly on unknown smooth functions of some predictor variables, and interest focuses on inference about these smooth functions. Therefore, the advantage of the generalized additive model (GAM) lies in relaxing the near universal statistical assumption of linearity, and thereby potentially allowing the discovery of important trends that may have been missed in traditional analyses. We actually tried to smooth all applicable continuous variables to see if non-linear relationship was expected. In the usual practice of assessing non-linearity, the value of a continuous variable is cut into ordinal strata and the ordinal variable is tested with a linear model. The smoothing technique takes segments of data and assesses the relationship of the continuous predictor with the outcome and gives a series of values rather than a single value for the beta estimate^26^. The equation of generalized additive model is as follows: g(E(y)) = β_0_ + f_1_(x_1_) + f_2_(x_2_) + ··· + f_m_(x_m_). The best fitted multivariate model was met by stepwise varible selection based on Akaike information criterion (AIC). First, we select the possible variables based on the conclusions of previous similar studies and univariate analysis between two groups. Then we applied stepwise model selection to looking for better fitted model. Last, we decided the final variables on the basis of statistical model selection based on Akaiki Information Criterion (AIC) and suggestion from other studies.

### Ethical consideration

This study was conducted according to the World Medical Association Declaration of Helsinki and conformed to ICMJE Recommendations for the Conduct, Reporting, Editing, and Publication of Scholarly Work in Medical Journals. This study was approved by the Ethical Committee of the Faculty of Medicine, Prince of Songkla University. Since the data were collected from the process of routine health examination, the content form was not required from the participants. Written informed consent was waived by the Ethical Committee of the Faculty of Medicine, Prince of Songkla University. All the information that might violate the right of privacy were not included in this study.

## Data Availability

The data that support the findings of this study are available from the corresponding author upon reasonable request.
